# Human papillomavirus infections among women with cervical lesions and cervical cancer in Eastern China: genotype-specific prevalence and attribution

**DOI:** 10.1186/s12879-017-2223-1

**Published:** 2017-01-31

**Authors:** Lei Zhang, Qingqing Bi, Hua Deng, Jing Xu, Juan Chen, Meilian Zhang, Xiaofeng Mu

**Affiliations:** 1 Department of Laboratory Medicine, Qingdao Central Hospital, Second Affiliated Hospital of Qingdao University, Siliunan Road #127, Qingdao, 266042 China; 2 Department of Pathology, Qingdao Central Hospital, Qingdao, China; 3 Clinical Laboratory, Qingdao Cancer Hospital, Qingdao, China

**Keywords:** Human papillomavirus, Genotypes, Attribution, Cervical cancer

## Abstract

**Background:**

Cervical cancer and its precursor, high-grade cervical intraepithelial neoplasia (CIN2/3), are associated with persistent high-risk human papillomavirus (HPV) infection. HPV genotype prevalence varies with severity of cervical lesions, patient age and geographical location. The aim of this study was to investigate HPV genotypes prevalence and attribution according to the severity of cervical lesions among Chinese women.

**Method:**

A 4-year surveillance study was performed. A total of 1664 female patients were included and their cervical histological diagnosis consisted of cervical intraepithelial neoplasia grade 1 (CIN1, 376 cases), grade 2 (CIN2, 408 cases), grade 3 (CIN3, 336 cases) and invasive cervical cancers (ICC, 544 cases). HPV genotypes prevalence and attribution to cervical lesions were calculated and analyzed. The 95% confidence interval (CI) for proportion was also calculated.

**Results:**

HPV positivity rates increased directly with cervical lesions severity (72.4% for CIN1, 81.4% for CIN2, 88.1% for CIN3 and 90.4% for ICC). Infections with multiple HPV types were inversely related to cervical lesions severity. HPV16, 52, 31, 33 and 58 were the most prevalent genotypes in ICC. 49.1% of squamous cell carcinoma, 65.1% of adenocarcinoma and 12.0–43.3% of cervical intraepithelial neoplasia could be attributed to vaccine-covered high-risk genotypes (HPV16/18). Inclusion of HPV52 and HPV31 in future vaccines would provide the highest marginal benefit in protection for individuals residing in this region.

**Conclusions:**

These findings provide information about HPV genotypes in this region which may be important to target with future vaccination and screening programs.

**Electronic supplementary material:**

The online version of this article (doi:10.1186/s12879-017-2223-1) contains supplementary material, which is available to authorized users.

## Background

Cervical intraepithelial neoplasia (CIN) is a premalignant lesion that is diagnosed by histology as CIN1, CIN2, or CIN3. If left untreated, CIN2 or CIN3 (high-grade CIN) might progress to cervical cancer. There were an estimated 527,600 new cervical cancer cases and 265,700 deaths worldwide in 2012 [1]. It is the second most commonly diagnosed cancer and third leading cause of cancer-induced death among females in less developed countries [1]. Persistent infection with one or more of carcinogenic genotypes of human papillomavirus (HPV) is a high-risk cause for cervical neoplasia [2]. At least 13 distinct high-risk HPV genotypes (HPV16, 18, 31, 33, 35, 39, 45, 51, 52, 56, 58, 59, and 68) have been identified to be associated with the risk of developing cervical cancer and defined as “carcinogenic” viral genotypes [3]. Carcinogenic HPV genotypes can be detected in more than 90% of cervical cancer specimens and in more than 60% of CIN tissues [4, 5].

For prevention of HPV infection and the potential development of cervical neoplasia, two prophylactic vaccines (Cervarix by GlaxoSmithKline and Gardasil by Merck Sharp and Dohme) are currently available. These two HPV vaccines potentially provide protection against two most common HPV genotypes, HPV16 and 18, which cause approximately 70% of invasive cervical cancers worldwide [6]. However, prevalence of the other carcinogenic HPV genotypes varies markedly by geographic region [5, 7]. In East Asia, HPV52 and 58 are more prevalent compared to worldwide [8]. Therefore, knowledge of the regional prevalence of HPV genotypes is essential for the development of effective vaccination as well as preventative screening strategies against cervical cancer.

A recent study performed by our group reported the HPV prevalence among female outpatients from Qingdao [9], which is a major city located in eastern China with a population of over nine million where prophylactic vaccines against HPV are currently not available. In the present study, we investigated the prevalence and attribution of HPV genotypes to cervical intraepithelial neoplasia and invasive cancer among women from this region.

## Methods

### Study subjects and specimen collection

From January 2012 to December 2015, women who visited gynecologists or oncologists seeking for further management of abnormal cervical cytology results in Qingdao Central Hospital and Qingdao Cancer Hospital, a tertiary hospital attached to Qingdao University with 1600 beds and a tertiary hospital specializing in oncology, were enrolled with informed consents. A participant was eligible if she (a) was sexually active, (b) was a gynecological patient, (c) was no previously miscarriage nor presently pregnant, (d) had not undergone a total hysterectomy, and (e) agreed to receive HPV genotyping and cervical histopathology evaluation. A cervical scrape sample was collected for HPV genotyping. Biopsy-confirmed histopathologic findings were used to define the status of cervical neoplasia. All biopsy samples were taken by a gynecologist according to guidelines from International Agency for Research on Cancer, WHO [10]. All procedures of this study were approved by Research Ethics Boards at Qingdao Central Hospital Affiliated to Qingdao University (Ethics 2013 No. 023), in accordance with the ethical standards of the Declaration of Helsinki.

### HPV genotyping and cervical histopathology

Cervical scrape samples were stored in a specimen transport medium. DNA extraction, amplification and hybridization were performed using an HPV genotyping kit (Yaneng Biotech, Shenzhen, China) according to the manufacturer’s instructions. The HPV DNA chip in the kit contained 23 type-specific probes that recognize 18 high-risk (16, 18, 31, 33, 35, 39, 45, 51, 52, 53, 56, 58, 59, 66, 68, 73, 82 and 83) and 5 low-risk HPV genotypes (6, 11, 42, 43 and 81). PCR amplifications were performed in a T100 Thermal Cycler (Bio-Rad, USA) with the following parameters: enzyme activation at 50 ^°^C for 15 min, initial denaturation at 95 ^°^C for 10 min, followed by 10 cycles of 94 ^°^C for 10 s, 42 ^°^C for 90 s and 72 ^°^C for 30 s, followed by 30 cycles of 94 ^°^C for 10 s, 46 ^°^C for 60 s and 72 ^°^C for 20 s, ending with a final extension at 72 ^°^C for 5 min. The PCR product (25 μl) was denatured at 95 °C for 10 min and immediately chilled on ice. Then, the HPV DNA hybridization reaction was performed on an automatic analyzer according to the manufacturer’s instructions. The hybridization procedure was presented in Additional file 1: Figure S1. The final results were obtained by colorimetric change on the chip under direct visualization. HPV negative and positive controls, provided in the kit, were simultaneously detected in every test.

The biopsy specimens obtained from the subjects were fixed by formalin, embedded in paraffin and stained with hematoxylin-eosin for histopathology in the pathology laboratory at Qingdao Central Hospital. Cervical histological diagnosis of the specimens was reported only when three different pathologists, from Qingdao Central Hospital and Qingdao Cancer Hospital, agreed with each other. Pathology results were classified into cervical intraepithelial neoplasia grade 1 (CIN1), CIN grade 2 (CIN2), CIN grade 3 (CIN3) and invasive cervical cancer (ICC) according to cervical lesions classification criteria [11].

### Statistical analysis

Frequencies of clinical data were calculated. The prevalence and attribution rates of HPV genotypes were calculated according to a previous method [8]. Crude prevalence was estimated by analyzing all studied cases as the denominator for every disease grade. The relative distribution for each disease grade was calculated only using the HPV positive samples as the denominator. The attribution of an HPV genotype to certain disease grade was calculated as follows: (percentage of samples with single-type infection) + (percentage of samples with multiple-type infection multiplied by attribution factor). The “attribution factor” for each HPV genotype was calculated based on the formula “number of samples with single-type infection of the HPV genotype concerned divided by the number of samples with single-type infection of any HPV genotype in that disease category”. The 95% confidence interval (CI) for proportion was calculated by the Wilson score method [12]. Differences in HPV positivity rates and proportions of multiple-type HPV infections between disease categories were assessed by Pearson’s *χ* ^2^ test and *p* < 0.05 was considered significant.

## Results

### HPV infection according to cervical pathology status

According to the inclusive criteria, a total of 1664 women received HPV genotyping and cervical histopathology evaluation. Their cervical pathology status were as follows: 376 cases of CIN1, 408 cases of CIN2, 336 cases of CIN3, 440 cases of squamous cell carcinoma (SCC) and 104 cases of adenocarcinoma. HPV positivity rates and the proportion of single- and multiple-type infection for each cervical pathology status are presented in Table 1. HPV positivity rates of CIN1, CIN2, CIN3 and SCC samples were 72.4, 81.4, 88.1 and 94.5%, respectively, which increased directly with severity of cervical lesions. Further, the HPV positivity rate of CIN1 group was significantly lower than that of invasive cervical cancer group (*p* = 6.36 × 10^-13^). The HPV positivity rate of adenocarcinoma group was significantly lower than squamous cell carcinoma group (*p* = 2.13 × 10^-11^). In addition, patients with lower grade cervical lesions (CIN1, 42.6%) displayed a higher frequency of multiple-type HPV infections compared with those suffering from higher grade cervical lesions (CIN2 and CIN3, 29.3%, *p* = 9.59 × 10^-5^) and invasive cancers (15.4%, *p* = 1.05 × 10^-16^).

**Table 1:** HPV infection according to cervical pathology status

Cervical Pathology status (total no. diagnosed)	HPV positive rate no. (%)	Single-type infection no. (%)	Multiple-type infection no. (%)		
			Two HPV genotypes	Three HPV genotypes	Four or more HPV genotypes
Cervical intraepithelial neoplasia (CIN)					
CIN grade 1 (376)	272 (72.4)	156 (41.5)	68 (18.1)	36 (9.6)	12 (3.2)
CIN grade 2 (408)	332 (81.4)	208 (51.0)	80 (19.6)	24 (5.9)	20 (4.9)
CIN grade 3 (336)	296 (88.1)	236 (70.2)	40 (11.9)	20 (6.0)	0
Invasive cervical cancer					
Squamous cell carcinoma (440)	416 (94.5)	348 (79.1)	60 (13.6)	8 (1.8)	0
Adenocarcinoma (104)	76 (73.1)	68 (65.4)	8 (7.7)	0	0

### Relative distribution of HPV genotypes according to cervical pathology status

**Fig. 1:**
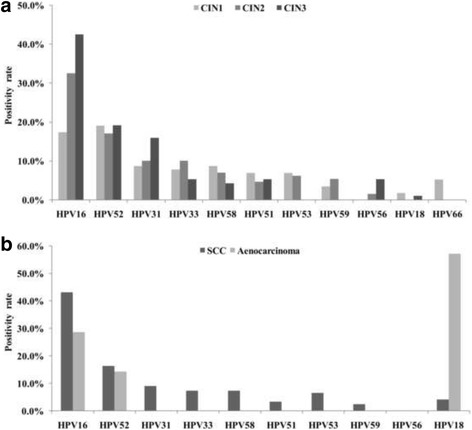
Relative distribution of HPV genotypes among different grades of cervical intraepithelial neoplasia (**a**) and invasive cervical cancers (**b**). Each kind of genotype was counted separately in cases with multiple-type HPV infections. The positivity rates of genotypes were calculated using the number of HPV positive samples in each disease grade as the denominator. CIN1, cervical intraepithelial neoplasia grade 1; CIN2, CIN grade 2; CIN3, CIN grade 3. SCC, squamous cell carcinoma

Detailed HPV genotypes distribution according to the severity of cervical lesions was also investigated. Each kind of genotype was counted separately in cases with multiple-type HPV infections. As shown in Fig. 1, distribution of the 11 most frequent genotypes in this study was presented. Top five most frequent genotypes among high-grade cervical lesions (both CIN2 and CIN3) were HPV16 (336/37.7%), HPV52 (160/17.9%), HPV31 (112/12.6%), HPV33 (72/8.1%) and HPV58 (52/5.8%). In contrast, the genotypes distribution was shown to be different in CIN1, where HPV52 was the most frequent genotype detected (88/19.1%), followed by HPV16 (80/17.4%) and HPV58 (40/8.7%) (Fig.1a). As shown in Fig.1b, among squamous cell carcinoma cases, HPV16 was the most common genotype (212/43.1%), followed by HPV52 (80/16.3%), HPV31 (44/8.9%) and HPV58 (36/7.3%). As for adenocarcinoma, only three kinds of genotypes were detected and HPV18 was the most prevalent type (48/57.1%), followed by HPV16 (24/28.6%) and HPV52 (12/14.3%).

### Attribution of HPV genotypes in cervical lesions

The crude prevalence and attribution rates of all HPV genotypes to each cervical pathology status were presented in Table 2. The majority (44.6%) of squamous cell carcinoma was attributed to HPV16. Subsequently, 15.2, 5.6 and 4.5% of squamous cell carcinoma were attributed to HPV52, HPV53 and HPV18, respectively. In contrast, the majority (44.8%) of adenocarcinoma was attributed to HPV18 and the following genotypes were HPV16 (20.3%) and HPV52 (4.2%).

**Table 2:** Prevalence and attribution of all HPV genotypes according to cervical pathology status

HPV Geno-types		Crude prevalence and attribution in % (95% confidence interval)				
		Squamous cell carcinoma (440)	Adeno-carcinoma (104)	CIN3 (336)	CIN2 (408)	CIN1 (376)
HPV16	Prevalence	48.2 (43.5–52.8)	23.1 (16.0–32.0)	47.6 (42.3–53.0)	41.2 (36.5–46.0)	21.3 (17.4–25.7)
	Attribution	44.6 (40.0–49.3)	20.3 (13.7–29.0)	43.3 (38.1–48.6)	30.7 (26.4–35.4)	9.9 (7.3–13.3)
HPV52	Prevalence	18.2 (14.9–22.1)	11.5 (6.7–19.1)	21.4 (17.4–26.1)	21.6 (17.9–25.8)	23.4 (19.4–27.9)
	Attribution	15.2 (12.1–18.8)	4.2 (1.7–10.0)	11.1 (8.2–14.9)	14.1 (11.0–17.8)	18.8 (15.2–23.1)
HPV31	Prevalence	10.0 (7.5–13.2)	0.0 (0.0–3.6)	17.9 (14.1–22.3)	12.7 (9.9–16.3)	10.6 (7.9–14.2)
	Attribution	3.9 (2.4–6.1)	0.0 (0.0–3.6)	10.6 (7.8–14.4)	8.6 (6.2–11.7)	3.8 (2.3–6.2)
HPV33	Prevalence	8.2 (6.0–11.1)	0.0 (0.0–3.6)	6.0 (3.9–9.0)	12.7 (9.9–16.3)	9.6 (7.0–13.0)
	Attribution	1.1 (0.5–2.6)	0.0 (0.0–3.6)	3.7 (2.2–6.3)	2.4 (1.3–4.4)	5.9 (3.9–8.7)
HPV58	Prevalence	8.2 (6.0–11.1)	0.0 (0.0–3.6)	4.8 (3.0–7.6)	8.8 (6.4–12.0)	10.6 (7.9–14.2)
	Attribution	3.8 (2.4–6.0)	0.0 (0.0–3.6)	3.7 (2.1–6.2)	3.2 (1.9–5.4)	3.8 (2.3–6.2)
HPV51	Prevalence	3.6 (2.3–5.8)	0.0 (0.0–3.6)	6.0 (3.9–9.0)	5.9 (4.0–8.6)	8.5 (6.1–11.8)
	Attribution	2.7 (1.6–4.7)	0.0 (0.0–3.6)	0.0 (0.0–1.1)	0.0 (0.0–0.9)	0.0 (0.0–1.0)
HPV53	Prevalence	7.3 (5.2–10.1)	0.0 (0.0–3.6)	0.0 (0.0–1.1)	7.8 (5.6–10.9)	8.5 (6.1–11.8)
	Attribution	5.6 (3.8–8.2)	0.0 (0.0–3.6)	0.0 (0.0–1.1)	4.2 (2.6–6.6)	0.0 (0.0–1.0)
HPV59	Prevalence	2.7 (1.6–4.7)	0.0 (0.0–3.6)	0.0 (0.0–1.1)	6.9 (4.8–9.7)	4.3 (2.6–6.8)
	Attribution	2.7 (1.5–4.7)	0.0 (0.0–3.6)	0.0 (0.0–1.1)	0.0 (0.0–0.9)	3.3 (1.9–5.6)
HPV56	Prevalence	0.0 (0.0–0.9)	0.0 (0.0–3.6)	6.0 (3.9–9.0)	2.0 (1.0–3.8)	0.0 (0.0–1.0)
	Attribution	0.0 (0.0–0.9)	0.0 (0.0–3.6)	6.0 (3.9–9.1)	0.0 (0.0–0.9)	0.0 (0.0–1.0)
HPV35	Prevalence	0.9 (0.4–2.3)	0.0 (0.0–3.6)	0.0 (0.0–1.1)	0.0 (0.0–0.9)	6.4 (4.3–9.3)
	Attribution	0.0 (0.0–0.9)	0.0 (0.0–3.6)	0.0 (0.0–1.1)	0.0 (0.0–0.9)	0.0 (0.0–1.0)
HPV18	Prevalence	4.5 (3.0–6.9)	46.2 (36.9–55.7)	1.2 (0.5–3.0)	0.0 (0.0–0.9)	2.1 (1.1–4.1)
	Attribution	4.5 (2.9–6.9)	44.8 (35.6–54.3)	0.0 (0.0–1.1)	0.0 (0.0–0.9)	2.1 (1.1–4.1)
HPV45	Prevalence	0.0 (0.0–0.9)	0.0 (0.0–3.6)	0.0 (0.0–1.1)	2.0 (1.0–3.8)	0.0 (0.0–1.0)
	Attribution	0.0 (0.0–0.9)	0.0 (0.0–3.6)	0.0 (0.0–1.1)	0.0 (0.0–0.9)	0.0 (0.0–1.0)
HPV68	Prevalence	0.0 (0.0–0.9)	0.0 (0.0–3.6)	0.0 (0.0–1.1)	0.0 (0.0–0.9)	4.3 (2.6–6.8)
	Attribution	0.0 (0.0–0.9)	0.0 (0.0–3.6)	0.0 (0.0–1.1)	0.0 (0.0–0.9)	0.0 (0.0–1.0)
HPV73	Prevalence	0.0 (0.0–0.9)	0.0 (0.0–3.6)	1.2 (0.5–3.0)	0.0 (0.0–0.9)	0.0 (0.0–1.0)
	Attribution	0.0 (0.0–0.9)	0.0 (0.0–3.6)	0.0 (0.0–1.1)	0.0 (0.0–0.9)	0.0 (0.0–1.0)
HPV82	Prevalence	0.0 (0.0–0.9)	0.0 (0.0–3.6)	0.0 (0.0–1.1)	4.9 (3.2–7.4)	3.2 (1.8–5.5)
	Attribution	0.0 (0.0–0.9)	0.0 (0.0–3.6)	0.0 (0.0–1.1)	0.0 (0.0–0.9)	0.0 (0.0–1.0)
HPV66	Prevalence	0.0 (0.0–0.9)	0.0 (0.0–3.6)	0.0 (0.0–1.1)	0.0 (0.0–0.9)	6.4 (4.3–9.3)
	Attribution	0.0 (0.0–0.9)	0.0 (0.0–3.6)	0.0 (0.0–1.1)	0.0 (0.0–0.9)	1.2 (0.5–2.9)
HPV39	Prevalence	0.0 (0.0–0.9)	0.0 (0.0–3.6)	0.0 (0.0–1.1)	0.0 (0.0–0.9)	2.1 (1.1–4.1)
	Attribution	0.0 (0.0–0.9)	0.0 (0.0–3.6)	0.0 (0.0–1.1)	0.0 (0.0–0.9)	0.0 (0.0–1.0)
HPV83	Prevalence	0.0 (0.0–0.9)	0.0 (0.0–3.6)	0.0 (0.0–1.1)	0.0 (0.0–0.9)	0.0 (0.0–1.0)
	Attribution	0.0 (0.0–0.9)	0.0 (0.0–3.6)	0.0 (0.0–1.1)	0.0 (0.0–0.9)	0.0 (0.0–1.0)
HPV81	Prevalence	0.0 (0.0–0.9)	0.0 (0.0–3.6)	0.0 (0.0–1.1)	0.0 (0.0–0.9)	1.1 (0.4–2.7)
	Attribution	0.0 (0.0–0.9)	0.0 (0.0–3.6)	0.0 (0.0–1.1)	0.0 (0.0–0.9)	0.0 (0.0–1.0)
HPV6	Prevalence	0.0 (0.0–0.9)	0.0 (0.0–3.6)	0.0 (0.0–1.1)	0.0 (0.0–0.9)	0.0 (0.0–1.0)
	Attribution	0.0 (0.0–0.9)	0.0 (0.0–3.6)	0.0 (0.0–1.1)	0.0 (0.0–0.9)	0.0 (0.0–1.0)
HPV43	Prevalence	0.0 (0.0–0.9)	0.0 (0.0–3.6)	0.0 (0.0–1.1)	0.0 (0.0–0.9)	0.0 (0.0–1.0)
	Attribution	0.0 (0.0–0.9)	0.0 (0.0–3.6)	0.0 (0.0–1.1)	0.0 (0.0–0.9)	0.0 (0.0–1.0)
HPV11	Prevalence	0.0 (0.0–0.9)	0.0 (0.0–3.6)	0.0 (0.0–1.1)	0.0 (0.0–0.9)	0.0 (0.0–1.0)
	Attribution	0.0 (0.0–0.9)	0.0 (0.0–3.6)	0.0 (0.0–1.1)	0.0 (0.0–0.9)	0.0 (0.0–1.0)

HPV16 also accounted for the majority of high-grade lesions (CIN3 and CIN2) with attribution rate of 43.3 and 30.7%, respectively. HPV52 was the following genotype, which attributed to 11.1 and 14.1% of CIN3 and CIN2, respectively (Table 2). In contrast, HPV16 was not the most common genotype found in CIN1. Either by crude prevalence rate or attribution rate, HPV52 ranked higher than HPV16 in CIN1.

**Fig. 2:**
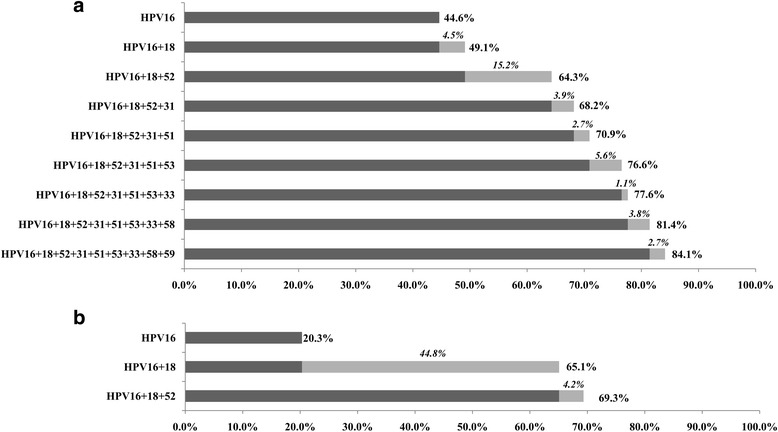
Cumulative attribution rates of high-risk HPV genotypes to invasive cervical cancer. **a** Squamous cell carcinoma; **b** adenocarcinoma. Marginal attribution rates conferred by individual HPV genotypes are italicized. Bold numbers represent cumulative attribution rates

**Fig. 3:**
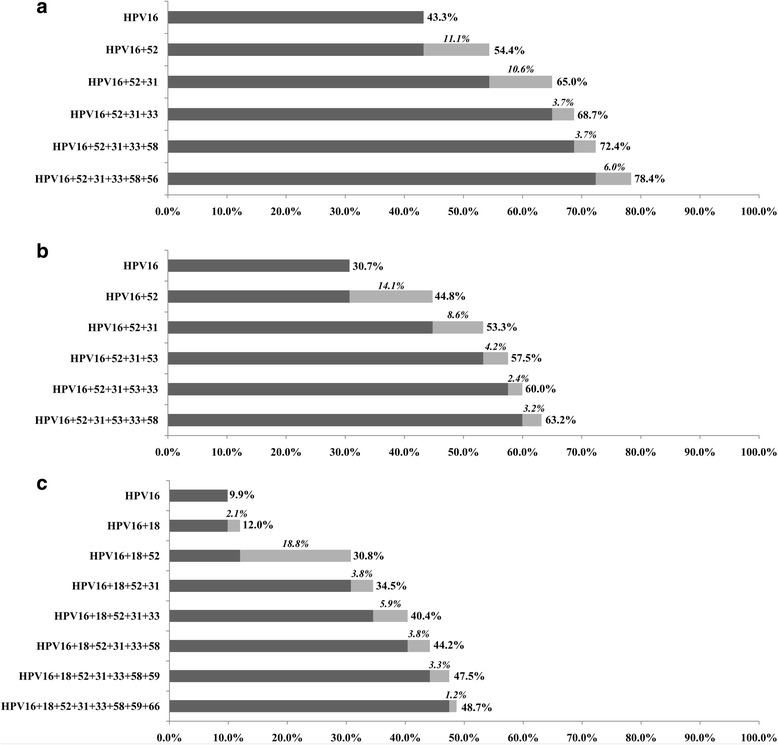
Cumulative attribution rates of high-risk HPV genotypes to cervical intraepithelial neoplasia. **a** CIN grade 3; **b** CIN grade 2; **c** CIN grade 1. Marginal attribution rates conferred by individual HPV genotypes are italicized. Bold numbers represent cumulative attribution rates

The cumulative attribution rates of 11 high-risk HPV genotypes to invasive cervical cancer and cervical intraepithelial neoplasia were presented in Figs. 2 and 3, respectively. The two high-risk types (HPV16 and HPV18) attributed to 49.1% of squamous cell carcinoma (Fig.2a), 65.1% of adenocarcinoma (Fig.2b), 43.3% of CIN3 (Fig.3a), 30.7% of CIN2 (Fig.3b) and 12.0% of CIN1 (Fig.3c) cases. The subsequent genotype, HPV52, brought the biggest marginal increase with 15.2% for squamous cell carcinoma (Fig.2a), 11.1% for CIN3, 14.1% for CIN2 and 18.8% for CIN1 lesions.

### Age-specific prevalence of cervical lesions and cancers

**Fig. 4:**
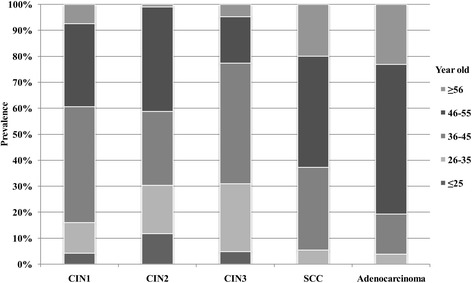
Age-specific prevalence of cervical lesions and cancers

All 1664 patients involved were classified into five groups according to age (≤25, 26–35, 36–45, 46–55, ≥56) and age-specific prevalence of cervical lesions and cancers was presented in Fig. 4. As shown in Fig. 4, 80.8% of the patients with adenocarcinoma and 62.7% with SCC were more than 46 years old. Moreover, 30.6% of the patients with high-grade cervical lesions (CIN2 and CIN3) were younger than 35.

## Discussion

Data of HPV prevalence and attribution according to cervical pathology status presented in this study may provide guidance for HPV vaccination program and preventative strategies against cervical cancer in this region of China. Among the involved female patients, HPV positivity rates increase directly with the cervical lesion severity, which is consistent with some previous studies from different regions of China [8, 13–15]. The overall HPV prevalence among ICC patients reaches 90.4% in this study, which is consistent with the worldwide HPV prevalence in cervical cancers, ranging from 86 to 94% [4, 16, 17]. In this study, single-type HPV infection is more prevalent in high-grade CIN and ICC patients than in low-grade CIN and the single-type HPV positivity rates also increase directly with the cervical lesion severity, which is consistent with a previous study from Beijing, China [18]. In this study, HPV infections are all high-risk genotypes among the high-grade CIN and ICC patients and the most prevalent genotypes are HPV16, 52, 31, 33 and 58. Another study in China reported that the most common HPV genotypes in high-grade CIN and cervical cancers are HPV16, 58, 31, 52, 33 and HPV16, 33, 18, 52, 58, respectively [13]. A multiple-center study in Beijing reported that HPV16, 58, 33, 56, and 31 are the most prevalent genotypes in cervical lesions [19]. It’s observed from these results that HPV16, 52, 31, 33, 58, 56 and 18 are prevalent genotypes among women with cervical lesions in China. A worldwide meta-analysis also confirmed that eastern Asia exhibits an HPV genotype distribution pattern distinct from the other areas of the world, a relatively high contribution of HPV52 and 58 to cervical lesions [20]. In this study, HPV18 was the most prevalent genotype among adenocarcinoma patients. This is in accordance with several studies focusing on cervical adenocarcinoma. HPV18 highly correlated to adenocarcinoma worldwide including China [21, 22]. Furthermore, HPV18, 16 and 45 have been reported to be the most prevalent genotypes in adenocarcinoma worldwide [20, 23]. However, in the present study, the presence of HPV52 instead of HPV45 in adenocarcinoma was observed and consistent with studies in eastern Asia [8, 20].

In this study, we found a high rate of young women with high grade cervical lesions (CIN2 and CIN3). 30.6% of patients with high-grade cervical lesions were younger than 35. Notably, 5.0% of patients with high-grade cervical lesions were even younger than 25, which is higher than that from a neighboring area of this region (3.4%) [24]. Therefore, for this region, vaccination program and preventative screening strategies against cervical cancer are expected to pay more attention to young women.

Furthermore, an approach adopted from previous studies [8, 25], which assumed a fractional attribution of individual HPV genotypes found simultaneously in a lesion, was applied to the current data. It was estimated that the current HPV16/18-based vaccines would protect against 49.1% of squamous cell carcinoma, 65.1% of adenocarcinoma and 12.0–43.3% of CINs. In this region, HPV52 and HPV31 were determined to be the next common types in contributing to squamous cell carcinoma and CINs, which is in accordance with several studies in eastern Asia including China [20, 26, 27]. Therefore, for this region of China, vaccination program and preventative strategies against cervical cancer are expected to pay attention to HPV52, 31, 58 and 33 along with vaccine preventable HPV 16 and 18. The two prophylactic vaccines mentioned, proved to be effective in preventing cervical cancer, are available in many countries worldwide except mainland China. Even if the 2-valent (HPV16 and 18) or 4-valent (HPV6, 11, 16 and 18) is going to be commercial available in mainland China, the demand for vaccines against more particular genotypes is still strong. The newcome 9-valent HPV vaccine may provide more protections against cervical lesions for women in this region of China.

In this present work, the “attribution factor” of each HPV genotype was calculated and cumulative attribution rates of prevalent high-risk HPV genotypes were presented. The 95% confidence interval (CI) for proportion was also calculated. These methods have been rarely used to analyze HPV infections in mainland China before. There are still some limitations in this study. The total number of samples involved in this study is not large. Multi-center study, including over three tertiary hospitals, may be more valuable. In addition, interactions between different HPV genotypes still need further investigation.

## Conclusions

This study presented HPV genotypes prevalence and attribution data among female patients with cervical lesions in this area, where the planning of HPV vaccination should be taken into account the prevalent genotypes of HPV16, HPV52, HPV31, HPV58 and HPV33. These data added new evidence that eastern Asia, including eastern China, exhibited an HPV genotype distribution pattern distinct from other parts of the world. Our results also could provide necessary reference for formulating a regional HPV-based screening strategy in the future.

## Additional file

Additional file 1: Figure S1. Reactions of HPV DNA hybridization, which were performed in an automatic analyzer. (DOC 125 kb)

## Abbreviations

CIN: Cervical intraepithelial neoplasia
HPV: Human papillomavirus
ICC: Invasive cervical cancer
SCC: Squamous cell carcinoma

## References

[1] Torre LA, Bray F, Siegel RL, Ferlay J, Lortet-Tieulent J, Jemal A (2015). Global Cancer Statistics, 2012. CA Cancer J Clin.

[2] Schiffman M, Castle PE, Jeronimo J, Rodriguez AC, Wacholder S (2007). Human papillomavirus and cervical cancer. Lancet.

[3] Schiffman M, Solomon D (2013). Clinical practice. Cervical-cancer screening with human papillomavirus and cytologic cotesting. N Engl J Med.

[4] Smith JS, Lindsay L, Hoots B, Keys J, Franceschi S, Winer R (2007). Human papillomavirus type distribution in invasive cervical cancer and high-grade cervical lesions: a meta-analysis update. Int J Cancer.

[5] Li N, Franceschi S, Howell-Jones R, Snijders PJ, Clifford GM (2011). Human papillomavirus type distribution in 30,848 invasive cervical cancers worldwide: Variation by geographical region, histological type and year of publication. Int J Cancer.

[6] Roden R, Wu TC (2006). How will HPV vaccines affect cervical cancer?. Nat Rev Cancer.

[7] Vaccarella S, Franceschi S, Zaridze D, Poljak M, Veerus P, Plummer M (2016). Preventable fractions of cervical cancer via effective screening in six Baltic, central, and eastern European countries 2017-40: a population-based study. Lancet Oncol.

[8] Chan PK, Cheung TH, Li WH, Yu MY, Chan MY, Yim SF (2012). Attribution of human papillomavirus types to cervical intraepithelial neoplasia and invasive cancers in southern China. Int J Cancer.

[9] Bi Q, Zhang L, Zhao Z, Mu X, Zhang M, Wang P (2015). Human papillomavirus prevalence and genotypes distribution among female outpatients in Qingdao, East China. J Med Virol.

[10] Sellors JW, Sankaranarayanan R (2003). Colposcopy and treatment of cervical intraepithelial neoplasia: a beginner’s manual.

[11] Sattar HA, Kumar V, Abbas AK, Aster JC (2013). Female Genital System and Breast. Robbins Basic Pathology.

[12] Newcombe RG (1998). Two-sided confidence intervals for the single proportion: comparison of seven methods. Stat Med.

[13] Ding X, Liu Z, Su J, Yan D, Sun W, Zeng Z (2014). Human papillomavirus type-specific prevalence in women referred for colposcopic examination in Beijing. J Med Virol.

[14] Liu X, Fan X, Yu Y, Ji L, Yan J, Sun AH (2014). Human papillomavirus prevalence and type-distribution among women in Zhejiang Province, Southeast China: a cross-sectional study. BMC Infect Dis.

[15] Zhang WY, Xue YZ, Chen M, Han L, Luo M (2008). Prevalence of high-risk human papillomavirus infection in different cervical lesion among organized health-examination women in Shanghai, China. Chin Med J (Engl).

[16] de Sanjose S, Quint WG, Alemany L, Geraets DT, Klaustermeier JE, Lloveras B (2010). Human papillomavirus genotype attribution in invasive cervical cancer: A retrospective cross-sectional worldwide study. Lancet Oncol.

[17] Forman D, de Martel C, Lacey CJ, Soerjomataram I, Lortet-Tieulent J, Bruni L (2012). Global burden of human papillomavirus and related diseases. Vaccine.

[18] Li Y, Huang K, Ji PL, Song L, Liu HT (2016). Cervical infection of oncogenic human papillomavirus (HPV) types in Beijing, China. Biomed Environ Sci.

[19] Li C, Wu M, Wang J, Zhang S, Zhu L, Pan J (2010). A population-based study on the risks of cervical lesion and human papillomavirus infection among women in Beijing, People’s Republic of China. Cancer Epidemiol Biomarkers Prev.

[20] Chan PK, Ho WC, Chan MC, Wong MC, Yeung AC, Chor JS (2014). Meta-analysis on prevalence and attribution of human papillomavirus types 52 and 58 in cervical neoplasia worldwide. PLoS One.

[21] Berrington de Gonzalez A, Green J (2007). Comparison of risk factors for invasive squamous cell carcinoma and adenocarcinoma of the cervix: collaborative reanalysis of individual data on 8097 women with squamous cell carcinoma and 1374 women with adenocarcinoma from 12 epidemiological studies. Int J Cancer.

[22] Li J, Zhang D, Zhang Y, Wang X, Lin Y, Hu L (2011). Prevalence and genotype distribution of human papillomavirus in women with cervical cancer or high-grade precancerous lesions in Chengdu, western China. Int J Gynaecol Obstet.

[23] Pirog EC, Lloveras B, Molijn A, Tous S, Guimerà N, Alejo M (2014). HPV prevalence and genotypes in different histological subtypes of cervical adenocarcinoma, a worldwide analysis of 760 cases. Mod Pathol.

[24] Hu SY, Hong Y, Zhao FH, Lewkowitz AK, Chen F, Zhang WH (2011). Prevalence of HPV infection and cervical intraepithelial neoplasia and attitudes towards HPV vaccination among Chinese women aged 18–25 in Jiangsu Province. Chin J Cancer Res.

[25] Insinga RP, Liaw KL, Johnson LG, Madeleine MM (2008). A systematic review of the prevalence and attribution of human papillomavirus types among cervical, vaginal, and vulvar precancers and cancers in the United States. Cancer Epidemiol Biomarkers Prev.

[26] Sasagawa T, Maehama T, Ideta K, Irie T, Itoh F, J-HERS Study Group (2016). Population-based study for human papillomavirus (HPV) infection in women in Japan: A multicenter study by the Japanese human papillomavirus disease education research survey group (J-HERS). J Med Virol.

[27] Sun B, He J, Chen X, He M, He Z, Wang Y (2014). Prevalence and genotype distribution of human papillomavirus infection in Harbin, Northeast China. Arch Virol.

