# Supplementary figures and images for: Human papillomavirus infections among women with cervical lesions and cervical cancer in Eastern China: genotype-specific prevalence and attribution
Source: BMC Infect Dis. 2017 Jan 31;17:107. doi: 10.1186/s12879-017-2223-1 (PMC5282745; doi:10.1186/s12879-017-2223-1)

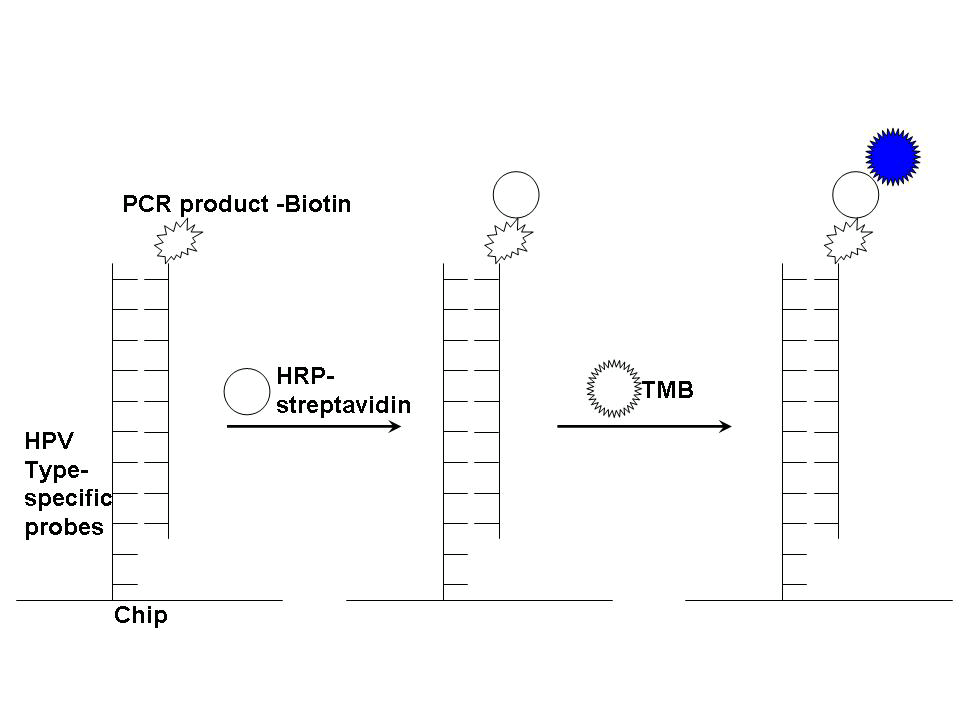


**Figure.S1.** **Reactions of HPV DNA hybridization, which were performed in an automatic analyzer.**

Supplement: Additional file 1: Figure S1. — Reactions of HPV DNA hybridization, which were performed in an automatic analyzer. (DOC 125 kb) [file 12879_2017_2223_MOESM1_ESM.doc]
